# Physical Exercise: A Novel Tool to Protect Mitochondrial Health

**DOI:** 10.3389/fphys.2021.660068

**Published:** 2021-04-27

**Authors:** Daniela Sorriento, Eugenio Di Vaia, Guido Iaccarino

**Affiliations:** ^1^Department of Advanced Biomedical Sciences, Federico II University of Naples, Naples, Italy; ^2^CIRIAPA Interdepartmental Center for Research on Arterial Hypertension and Associated Conditions, Federico II University of Naples, Naples, Italy

**Keywords:** energetic metabolism, mitochondrial dysfunction, cardiovascular disease, physical activity, heart

## Abstract

Mitochondrial dysfunction is a crucial contributor to heart diseases. Alterations in energetic metabolism affect crucial homeostatic processes, such asATP production, the generation of reactive oxygen species, and the release of pro-apoptotic factors, associated with metabolic abnormalities. In response to energetic deficiency, the cardiomyocytes activate the Mitochondrial Quality Control (MQC), a critical process in maintaining mitochondrial health. This process is compromised in cardiovascular diseases depending on the pathology’s severity and represents, therefore, a potential therapeutic target. Several potential targeting molecules within this process have been identified in the last years, and therapeutic strategies have been proposed to ameliorate mitochondria monitoring and function. In this context, physical exercise is considered a non-pharmacological strategy to protect mitochondrial health. Physical exercise regulates MQC allowing the repair/elimination of damaged mitochondria and synthesizing new ones, thus recovering the metabolic state. In this review, we will deal with the effect of physical exercise on cardiac mitochondrial function tracing its ability to modulate specific steps in MQC both in physiologic and pathologic conditions.

## Introduction

**FIGURE 1:**
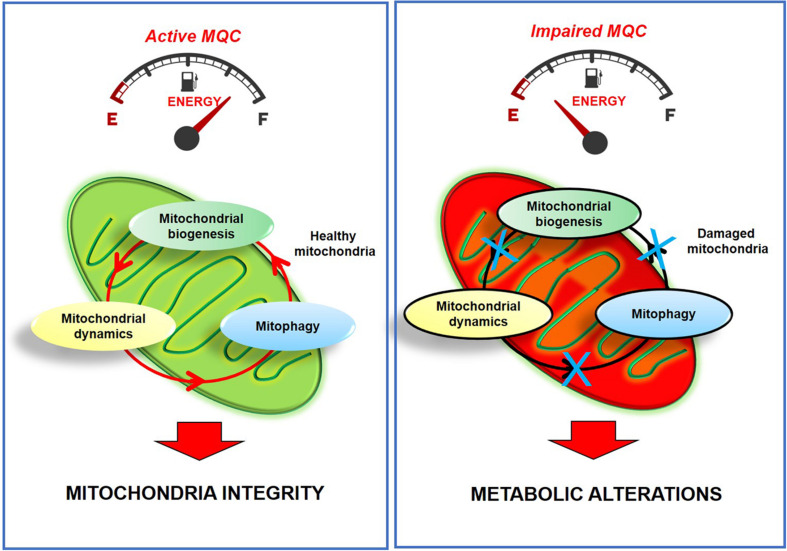
Active MQC is essential for maintaining mitochondria integrity and function. The impairment of MQC leads to metabolic alterations and irreversible mitochondria damage.

Mitochondria are considered the “energy power station” of the cells due to their ability to regulate energy metabolism. These organelles also regulate critical cellular processes, such as calcium homeostasis, and cell survival (Giorgi et al., 2018; Sprenger and Langer, 2019; Fan et al., 2020). Thus, their health is critical to maintaining wellness in organs and tissues (Youle and van der Bliek, 2012; Eisner et al., 2018), especially in high metabolic active tissues that need much energy to support their activities. In this context, cardiac cells should supply the heart’s large energy requests for its pumping activity. Therefore, mitochondria quality control is essential to avoid alterations in cardiac physiological processes such as ATP production, ROS generation, and survival/apoptotic mechanisms. To avoid metabolic alterations, mitochondria are carefully monitored through a complex process, called mitochondrial quality control (MQC; Ni et al., 2015). Such a process includes post-translational modification of mitochondrial proteins, mitochondrial dynamics, and autophagy (Fan et al., 2020). In response to stimuli, such as cardiotoxic drugs, ischemia/reperfusion, pressure overload, mitochondria health is compromised, favoring heart disease development, such as cardiac hypertrophy, dilated cardiomyopathy, ischemia/reperfusion injury, heart failure. Compensatory mechanisms are therefore activated by cardiac cells to favor cell survival (*mitochondrial dynamics*): the cleavage of the damaged parts of mitochondria and the fusion of healthy ones (*fission/fusion*), the elimination of irreversibly damaged mitochondria (*mitophagy*), and the replacement of lost mitochondria (*mitochondrial biogenesis*). Alterations in one of these steps lead to mitochondrial dysfunction, compromise cell metabolism, and trigger pathologic conditions (Figure 1). Several targeted approaches have been proposed to ameliorate mitochondrial function in failing hearts, including agonists for the PPARs and ERRs, SIRT1, and AMPK (Andreux et al., 2013). In this field, physical exercise is emerging as a non-pharmacologic tool to attenuate mitochondrial dysfunction in pathologic conditions, including cardiovascular diseases.

## The Molecular Mechanisms of Mitochondria Monitoring in the Heart

The pathogenesis of heart dysfunction is based on the activation of multiple and complex mechanisms. Among them, mitochondrial dysfunction is a common hallmark of cardiomyocyte damage (Bayeva et al., 2013; Ciccarelli et al., 2020). To support energetic heart demands, cardiac cells are rich in mitochondria (30% of the total cell volume) to provide adequate ATP supply. Therefore, in these cells, MQC is fundamental to ensure good mitochondrial homeostasis (Disatnik et al., 2013, 2015). In the heart, the distribution and metabolic function of mitochondria is associated with the myocardium’s developmental stage (Vasquez-Trincado et al., 2016). Indeed, in neonatal cardiomyocytes, energy derives mainly from glycolysis and glucose oxidation, mitochondria have a reticular distribution in the cytosol and can move freely within the cell (Lopaschuk and Jaswal, 2010; Vasquez-Trincado et al., 2016). In adult cardiomyocytes, energy oxidation of fatty acids is the primary energy source, mitochondria have interfibrillar, subsarcolemmal, and perinuclear localization and their movements are limited (Stanley et al., 2005; Vasquez-Trincado et al., 2016). Cardiac mitochondria are part of dynamic networks depending on the balance between fusion and fission processes and are relevant to several processes of cardiovascular biology, such as cardiac development, responses to ischemia/reperfusion injury, heart failure, and apoptosis (Vasquez-Trincado et al., 2016). In normal conditions, the levels of mitochondrial proteins involved in fusion should be high to support oxidative phosphorylation capacity (Youle and van der Bliek, 2012). Post-translational modifications of specific mitochondrial proteins, such as ubiquitination of Myro2, increased SUMOylation of DRP1, decreased SUMOylation of mitofusins (MFN2), phosphorylation of DRP1, or alterations in their expression levels, such as excessive increase or deficiency of PGC1-alpha, increased levels of DRP1, reduced expression of MFN2 or OPA-1, leads to several heart diseases (Fan et al., 2020). This evidence comes from studies in preclinical models based on the downregulation or knockout of specific mitochondrial genes, MFNs, optic atrophy 1 (OPA1), and DRP1 (Papanicolaou et al., 2011, 2012a,b; Piquereau et al., 2012; Sharp et al., 2014) proposing these proteins as potential targets to ameliorate cardiac function. Research in the field is still ongoing to better define the specific pathways that are active in the heart in response to stress.

### Mitochondria Fission and Fusion

The organelle’s morphology dictates the mitochondrial function: a critical feature in the MQC is the mitochondrial network structure’s dynamic nature. Through fission and fusion events, mitochondria continuously change their shape (from small puncta to interconnected networks), adapting to the energetic status and the different metabolic supplies (Otera and Mihara, 2011). Indeed, increasing mitochondrial fusion results in elongated mitochondria and the increase of network interconnectivity while increasing mitochondrial fission results in fragmented unconnected mitochondria (Dorn, 2015). The most representative subtypes of mitochondrial morphology include small spheres, swollen spheres, straight rods, twisted rods, branched rods, and loops (McCarron et al., 2013; Leonard et al., 2015). This classification is based on the analysis of specific measures (branch count, circularity, form factor, branch length, and mito-area) in images of mitochondria labeled with a specific fluorescent probe, Mito-tracker. This analysis of mitochondrial morphology is critical to identify defects in mitochondrial dynamics. Indeed, alterations in the mitochondrial network organization are classic features of many metabolic diseases, especially in their early stages (Galloway and Yoon, 2013). The molecular machinery that controls fusion and fission processes is finely regulated. Fusion is required to maintain mitochondrial DNA and cellular respiration (Chen et al., 2010; Sprenger and Langer, 2019) and is essential for embryonic development (Chen et al., 2003) and tissue homeostasis (Song et al., 2015b). This process is regulated by the mitofusins (MFN1 and MFN2) on the outer mitochondrial membranes and by OPA1 on the internal mitochondrial membranes (Cerveny et al., 2007). A recent study shows that MFNs change their conformations in response to specific intramolecular interactions and the targeting of these conformational changes can correct defects in mitochondrial dynamics (Franco et al., 2016), suggesting the critical role of MFNs. Mitochondrial fission is needed for inheritance and the removal of damaged mitochondria and is regulated by DRP1, a cytoplasmic GTPase that is recruited to mitochondria in response to stress (Taguchi et al., 2007). The genetic deletion of DRP1 in the heart blocks mitochondrial fission and upregulates Parkin, leading to lethal cardiomyopathy (Song et al., 2015a). Alterations in fission and fusion events mine mitochondrial function and represent a common feature in several human diseases (Archer, 2013).

### Autophagy/Mitophagy

Autophagy is the “cleaner” of the cell to remove dysfunctional proteins and organelles. In 2016 Prof. Yoshinori Ohsumi was awarded to Nobel Prize in Medicine for the identification of most proteins and pathways involved in the process (Tsukada and Ohsumi, 1993), the metabolic state sensors that regulate them (Eisner et al., 2018), and the fine mechanistic details of autophagosome formation (Nakatogawa et al., 2012). Three different autophagic mechanisms occur in mammals: microautophagy, chaperone-mediated autophagy (CMA), and macroautophagy (Wang and Klionsky, 2011; Shirakabe et al., 2016). Microautophagy allows the elimination of small portions of cytoplasm that are directly trapped through membrane invaginations of lysosomes. CMA determines a selective degradation of cytosolic proteins with particular sequences recognized by chaperones and transferred to lysosomes. Macroautophagy determines the degradation or recycling of proteins and organelles by trapping them in double-membrane structures (autophagosomes) that fuse with lysosomes. The degradation of the sequestered elements occurs through the activity of specific lysosomal hydrolases (Wang and Klionsky, 2011). The selective macroautophagy aimed to remove damaged mitochondria is called “mitophagy.” There are two central regulators of the autophagic process, mTOR, and AMPK (Kim et al., 2011). mTOR activity is inversely correlated with autophagy: when mTOR increases, autophagy shuts down (Dunlop and Tee, 2014). The mTOR complex 1 (mTORC1) activity is sensitive to fluctuations in amino acid levels. In amino acid-rich conditions, mTORC1 inactivates the autophagy initiators ULK (Rabanal-Ruiz et al., 2017). On the opposite, AMPK is a fine activator of autophagic processes (Dunlop and Tee, 2014). It is a sensor of intracellular energy through the detection of the AMP/ATP ratio. The increase of this latter activates the AMPK-dependent autophagic process to allow degradation or recycle of dysfunctional proteins and organelles (Tong et al., 2020). Also, protein acetylation seems to be involved in the regulation of autophagy in the heart, such as tubulin acetylation. Indeed, inhibiting tubulin deacetylation by histone deacetylase 6 reduced protein aggregates in cardiomyocytes and led to substantial improvement in cardiac function (McLendon et al., 2014). The analysis of autophagy is generally performed through the evaluation of the specific molecules involved in the autophagic machinery or evaluating the autophagic flux, that represents the measure of the autophagic degradation activity. The autophagic process includes the formation of the phagophore, the initial sequestering compartment, the completion of the autophagosome, the fusion with lysosomes and degradation of the contents. Defects in autophagic flux are evaluated through the detection of autophagosome turnover. Its accumulation indicates a block in fusion with lysosomes or disruption of lysosomal functions (Klionsky et al., 2012; Loos et al., 2014). Mitophagy, in particular, is a critical step in maintaining cardiac function at normal levels, and defects in such a process could trigger the metabolic alterations in cardiomyocytes. In the heart, it occurs through the activation of two different intracellular pathways: parkin dependent and independent mechanisms. The PINK1-Parkin axis is the most widely studied mitophagy pathway that is activated in response to mitochondrial membrane depolarization. In healthy mitochondria, transmembrane potential allows the import of PINK1 to the inner mitochondrial membrane where it is cleaved and degraded by the proteasome (Ding and Yin, 2012); thus, its levels are generally low. In damaged mitochondria, Pink levels increase inducing the recruitment and activation of Parkin (Kim et al., 2008; Ding and Yin, 2012), which in turn induces the ubiquitination of several mitochondrial proteins (MFN2, VDAC, and DRP1; Geisler et al., 2010). The adapter protein p62/SQSTM1 can promote Parkin-dependent mitophagy by interacting with both ubiquitin and LC3-II and favoring the mature autophagosome (Geisler et al., 2010). Autophagosome fusion with lysosomes allows the degradation of encapsulated materials by proteolytic enzymes (Vainshtein et al., 2015b). In this pathway, Parkin translocation to mitochondria and detection levels of LC3-II are considered specific markers of mitophagy. Autophagosome formation depends on the serine-threonine kinase ULK-1, which acts in complex with other proteins (Zachari and Ganley, 2017). In some cases, Parkin’s genetic deletion does not prevent mitophagy, which occurs through the activation of an alternative pathway independent of Parkin and protein ubiquitination. Indeed, in addition to the PINK1-Parkin pathway, other LC3-interacting proteins are also involved in mitophagy such as FUNDC1, BNIP3, or BNIP3L/NIX. They directly recruit autophagic machinery by a ubiquitin-independent mechanism to induce autophagosome formation in specific cell types (Liu et al., 2014). Four selective autophagy cargo receptors have been identified, p62 (SQSTM1), NBR1, NDP52, and Optineurin, which serve as mitophagy receptor in mammals. These receptors allow LC3-II binding and to specifically select mitochondria to be degraded into autophagosomes (Wang et al., 2019).

### Mitochondrial Biogenesis

Mitochondrial biogenesis is the process that regulates the synthesis of new mitochondria allowing the rescue of the mitochondrial mass to support the cardiac energy supplies (Scarpulla, 2011). This process is finely regulated by PGC1-alpha (Austin and St-Pierre, 2012; Dorn et al., 2015) which interacts with transcription factors (NRF1/2, ERR, and PPAR) and regulates the replication of mtDNA and the transcription of mitochondrial proteins genes (Dorn et al., 2015). The cardiac-specific overexpression of PGC1-alpha in mice increases mitochondrial biogenesis during the postnatal period (Lehman et al., 2000) while its genetic deletion has no effect under basal conditions but accelerates cardiac dysfunction in response to pressure overload (Arany et al., 2006). The germline deletion of PGC-1alpha induces a perinatal arrest of biogenesis and reduction in mitochondrial content (Lai et al., 2008). AMPK also regulates energy homeostasis directly, by phosphorylating metabolic enzymes and nutrient transporters, and indirectly, by promoting the transactivation of nuclear genes involved in mitochondrial biogenesis and function (Bergeron et al., 2001; Lai et al., 2008). Indeed, AMPK phosphorylates components of signaling pathways that enhance mitochondrial biogenesis such as PGC-1alpha (Bergeron et al., 2001). Also, it acts as an epigenetic regulator by phosphorylating three proteins involved in nucleosome remodeling, DNMT1, RBBP7, and HAT1 (Marin et al., 2017). Such phosphorylative events increase histone acetylation and decrease DNA methylation of PGC-1α, NRF1, NRF2, Tfam, UCP2, and UCP3 promoters (Marin et al., 2017) inducing mitochondrial biogenesis.

## Physical Training: Non-Pharmacological Therapy to Improve Human Health

Most people, mostly young, perform physical activity (PA) to lose weight and ameliorate their physical appearance. Besides these esthetic effects, PA emerged as a critical promoter of human health, especially in the presence of chronic pathologies. The American College of Sports Medicine generated guidelines and recommendations to direct toward a correct PA practice and the presence of complications (No authors listed, 1997; Kraemer et al., 2009; Schmitz et al., 2010; Garber et al., 2011). Therefore, a structured exercise training plan is now considered an integral part of the medical prescription for preventive and therapeutic purposes. Based on patients’ state of health and physical ability, a structured, personalized plan of exercise training can be prescribed, including the type and intensity of the exercises, duration, frequency, progression, and execution methods. The prescription of a good fitness program is fundamental to avoid injuries, and, in extreme cases, sudden death in athletes.

In healthy people, physical exercise induces several physiological changes to augment the cardiopulmonary system’s activity to deliver oxygen to all organs and tissues, including the heart. This action implicates several beneficial effects, especially in frail and non-frail older persons (Svantesson et al., 2015; Silva et al., 2017), favoring neuroplasticity and cognitive functions (Hotting and Roder, 2013), reducing stress (Bischoff et al., 2019), ameliorating physical performances and daily activities. Overall, PA is associated with a better quality of life and health outcomes, especially in elders. In part, physical performance depends on the composition of skeletal muscle since it includes different fiber types that are responsible for muscle plasticity in response to functional demands: slow oxidative fibers (type I) and fast glycolytic fibers (type II). Several stimuli can affect fiber-type switch, and PGC-1α seems to be the critical regulator of this phenomenon (Lin et al., 2002). This is not surprising, considering that PGC1-alpha induces mitochondrial biogenesis in different tissues and organs, contributing to mitochondrial energetics. Gene deletion of this protein in mice causes a shift from slow type I toward fast type II muscle fibers associated with exercise intolerance (reduced endurance capacity, fiber damage, and inflammation). In response to gene deletion, physical training increases total mitochondrial protein content within fibers (Lundby and Jacobs, 2016) and favors fiber type switch, by activating AMPK, the upstream regulator of PGC-1alpha (Rockl et al., 2007).

Physical activity also exerts beneficial effects in pathological conditions, such as childhood and adult obesity (Jakicic and Davis, 2011; Diaz Martinez et al., 2015), cancer (Jones et al., 2011, 2012), rheumatoid arthritis (Cooney et al., 2011), type 2 diabetes (Wilkerson et al., 2011), anthracyclines-induced cardiotoxicity (Scott et al., 2011), and cardiovascular diseases (Myers, 2003). In this context, the link between PA and cardiovascular diseases is becoming increasingly tight for the prevention and treatment of these conditions. Indeed, PA exerts beneficial effects on both cardiovascular risk and pathologies, as described below.

## Exercise Regulates Mitochondrial Phenotypes

**FIGURE 2:**
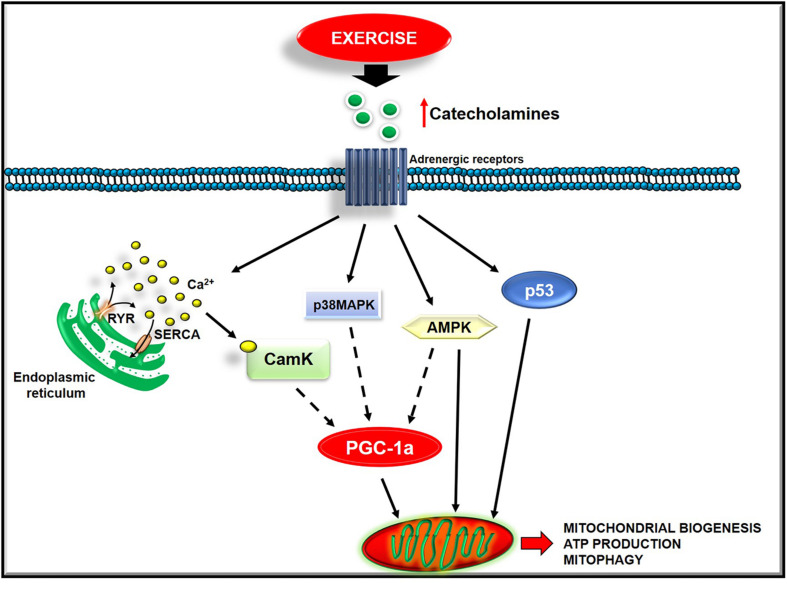
Exercise activates several intracellular pathways to regulate mitochondrial function.

Exercise triggers several changes in the mitochondrial dynamics and function that may be dependent upon exercise intensity. However, the precise mechanisms remain to be better elucidated and warrant future investigations. Below, we discuss the available findings on this issue (Figure 2).

### Exercise and Mitochondrial Biogenesis

Acute exercise activates several mechanisms that converge on PGC-1α, the master regulator of mitochondrial biogenesis, such as CaMK, p38 MAPK, AMPK, and p53 signaling. In L6 myotubes, an increase of cytosolic calcium induces PGC-1alpha, NRF-1, NRF-2, and mtTFA. This effect is prevented by both dantrolene, which blocks Ca^2+^ release from the SR, and a low concentration of the CAMK inhibitor, KN93 (Islam et al., 2020). These findings point to CaMK as a trigger of this signaling cascade (Islam et al., 2020). The activation of the p38-MAPK pathway also affects mitochondrial biogenesis by inducing PGC-1alpha promoter activity. Accordingly, through specific inhibitors or a dominant-negative form of p38, its inhibition exerts the opposite effect (Memme et al., 2019). The upstream activation of p38 MAPK signaling seems to be due to the increase of reactive oxygen species (Oliveira and Hood, 2019). AMPK activity also increases in response to exercise. This increase occurs in rats’ muscles running on a treadmill and in response to electrical stimulation (Belardinelli et al., 2012; Kachur et al., 2019). However, not all muscular adaptations to training are mediated by the activation of AMPK since this latter occurs in the superficial, white region of the quadriceps and soleus muscles of rats but not in the deep, red region of the quadriceps muscle (Belardinelli et al., 2012). Also, the tumor suppressor p53 is involved in the regulation of mitochondrial biogenesis (Ferreira et al., 2014). Indeed, preclinical studies show that its deletion reduces mitochondrial respiration and content, and endurance performance (Ferreira et al., 2014; Carter et al., 2015; Roh et al., 2016). In particular, p53 regulates both the mitochondrial transcription machinery, by translocating to mitochondria and activating TFAM (Lopez-Otin et al., 2013), and mitochondrial respiration, by interfering in the balance between glycolytic and oxidative pathways (Carter et al., 2015). The increase of intracellular calcium, ROS production, AMP/ATP ratio, circulating catecholamines are the upstream exercise signals that activate the above-described pathways (Fiorenza et al., 2018). The activation of these mechanisms also seems to be dependent on training intensity. Indeed, healthy men were asked to perform either sprint interval training (SIT), high−intensity interval training, or sub-lactate threshold continuous training for 4 weeks, and mitochondrial function was measured in muscle biopsy. The maximal mitochondrial respiration in muscle fibers increased significantly only after SIT and associates with a specific raised content of PGC-1alpha and p53 (Kim et al., 2017). Overall, these findings suggest that PA, based on training intensity, activates different intracellular pathways that favor new mitochondria synthesis.

### Exercise and Mitochondria Turnover

Physical activity also triggers cleaner processes to regulate the turnover of organelles: mitophagy and lysosomes biogenesis. Recent studies show that exercise improves mitochondrial quality and function by stimulating their turnover (Safdar et al., 2011; Cartee et al., 2016; Joseph et al., 2016). Acute exercise induces autophagy in skeletal and cardiac muscle of fed mice that is protective against metabolic disorders. Indeed, mice with knock-in mutations in BCL2 gene that prevent autophagy activation show decreased endurance and altered glucose metabolism during acute exercise (He et al., 2012). Exercise training promotes the degradation of abnormal mitochondria by autophagy, known as mitophagy (Vainshtein et al., 2015a; Laker et al., 2017; Yoshioka et al., 2019). Since AMPK is a known activator of autophagic flux and given the ability of exercise to induce its levels, PA likely induces mitochondrial turnover by activating AMPK dependent mechanisms.

### Exercise and Mitochondrial Morphology

Mitochondria morphology is severely affected in failing muscles, including the heart, and is a hallmark of mitochondrial dysfunction. This feature is finely regulated by fusion and fission processes (Cartoni et al., 2005; Ding et al., 2010). In this context, exercise affects mitochondrial morphology by activating specific molecular mechanisms. The muscle-specific gene Zmynd17 is known to control mitochondrial quality in muscle, especially in fast glycolytic muscles. Its deletion leads to abnormal mitochondria accumulation, whose number is significantly reduced after 10 weeks of voluntary exercise (Fujita et al., 2018). These findings underline that exercise’s beneficial effect occurs independently from Zmynd17 activity, suggesting the specificity of PA effects (Yoshioka et al., 2019). It has been shown that acute exercise increases mitofusins’ expression in human skeletal muscle and stimulates mitochondrial fusion by activating the PGC−1α/ERRα pathway (Cartoni et al., 2005). PGC1a overexpression in muscle leads to dense mitochondria with typical cristae structure and increases the endurance exercise capacity (Casuso and Huertas, 2020). This effect is also reproduced in humans. In highly trained swimmers subjected to two high-intensity swimming bouts, both SIT and HIT protocols induced mitochondrial fusion and increase MFN2 protein content (Huertas et al., 2019). Accordingly, DRP1 and MFN2 gene expression levels increase immediately following exercise (SIT and MICT) in healthy active subjects (Granata et al., 2017) and moderately trained subjects (Fiorenza et al., 2018). In response to high-intensity exercise, this effect also occurs and depends on β-adrenergic stimulation (Cribbs and Strack, 2007). Accordingly, preclinical studies show that acute exercise inhibits mitochondrial fission in a β-adrenergic-dependent manner and is mainly due to DRP1 inactivation through phosphorylation at Ser637 (Cribbs and Strack, 2007; Casuso and Huertas, 2020). PA regulates fission and fusion processes also affecting calcium handling. Indeed, HIT acutely induces ryanodine receptor 1 fragmentation, thus altering calcium uptake by the SR and increasing calcium release in the cytosol (Place et al., 2015). Altogether, these findings underline that exercise activates specific intracellular pathways to counteract the defects in mitochondrial dynamics.

### Exercise and Mitochondrial Respiration

Mitochondria are the primary source of ATP synthesis within the cell through the electron transport chain, and several factors could affect this activity, such as oxidative stress, nitric oxide, and substrate availability. Exercise can regulate mitochondrial respiration, thus affecting ATP production and mitochondrial function. Indeed, both acute and endurance exercise augments state four respiration and the respiratory control index (Han and Kim, 2013; Yoo et al., 2019).

### Exercise and Oxidative Stress

ROS are not necessarily detrimental but exert different effects depending on their levels. Physiological levels of ROS are essential to perform different cellular functions, such as the regulation of vascular tone by regulating nitric oxide synthase, the regulation of immune responses and apoptosis by activating specific transcription factors (AP-1 and NFkappaB), the regulation of insulin receptor kinase activity by activating protein tyrosine phosphatases (Fisher-Wellman and Bloomer, 2009). On the contrary, excessive amounts of ROS are pathologic and activate several molecular mechanisms leading to cell damage and death. ROS levels depends on the balance between its production and scavenging (Aon et al., 2010; Aon et al., 2012). Exercise can affect the oxidative state of the cell by increasing ROS production. It is not surprising given its ability to augment mitochondrial respiration, one of the primary sources of free radicals (Fisher-Wellman and Bloomer, 2009; Cooper et al., 2012). The heart has a high oxidative metabolic rate with scarce antioxidant activity and is, therefore, most sensitive to oxidative changes. Endurance training protects the heart from oxidative stress by upregulating both ROS, which themselves stimulate the redox system, and several antioxidant systems (Ascensao et al., 2003). However, depending on the mode, intensity, and duration of exercise, the amount of ROS could switch from a physiological to a pathological level determining the type of response from oxidative stress to adaptative responses.

## Cardiac Adaptative Responses to Exercise: The Physiological Cardiac Hypertrophy

In response to exercise-dependent hemodynamic stress of pressure and volume overload, the heart activates adaptative responses: metabolic remodeling and physiological hypertrophy. Physiological hypertrophy induced by exercise is characterized by a 10–20% increase of cardiac mass and normal or enhanced contractile function, at a non-pathologic level (Maillet et al., 2013). This effect is due to exercise dependent modulation of myocardial metabolism (fatty acid metabolism, carbohydrate metabolism, and mitochondrial adaptation). Indeed, exercise promotes fatty acid utilization through the up-regulation of carnitine acyltransferase shuttles (CPT-1 and CPT-2; Abel and Doenst, 2011). It dynamically regulates cardiac glucose utilization: in the acute phase, it reduces glycolysis by modulating phosphofructokinase activity favoring physiological cardiac remodeling; in the recovered phase, it increases myocardial phosphofructokinase activity and glycolysis (Gibb et al., 2017). This dynamic regulation of phosphofructokinase activity affects the glucose-fatty acid cycle and heart growth (Gibb et al., 2017). Exercise also promotes e-NOS dependent mitochondrial biogenesis (Vettor et al., 2014) and physiological ROS production (Alleman et al., 2014).

Furthermore, exercise-dependent physiological hypertrophy activates cardiac progenitor cells. Indeed, C-kit and Sca-1 positive cardiac stem cells, the main types of cardiac stem cells in the heart, are activated by swimming exercise training in mice (Xiao et al., 2014), which protects the heart in response to myocardial infarction and ischemia-reperfusion (IR) injury (Farah et al., 2013; Nicholson et al., 2013). Exercise also induces functional adaptation of the heart by improving cardiac function and cardiomyocyte contractile function by activating ryanodine receptors and sarcoendoplasmic reticulum Ca^2+^ ATPase SERCA (Wisloff et al., 2001; Esch et al., 2007; Kemi et al., 2008; Nystoriak and Bhatnagar, 2018).

## Exercise Reduces Cardiovascular Risk

**FIGURE 3:**
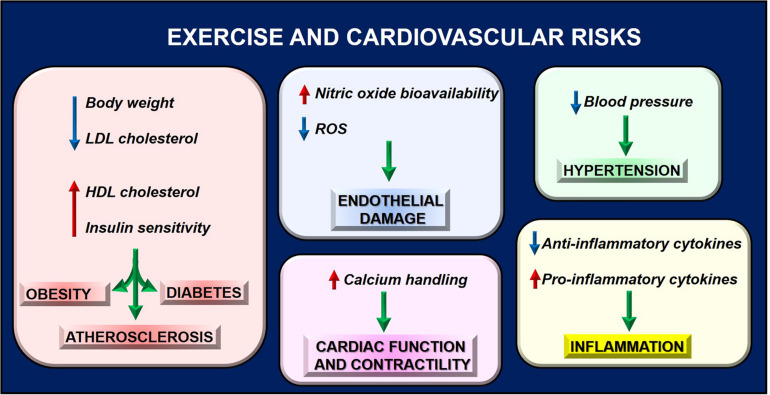
Exercise reduces cardiovascular risk by regulating several phenotypes, thus preventing pathologic conditions that contribute to cardiac dysfunction.

A sedentary lifestyle is considered a significant risk factor for cardiovascular disease while performing a regular PA could positively affect health. To date, exercise is considered a non-pharmacological intervention for improving cardiovascular fitness in healthy and diseased individuals increasing exercise tolerance and ameliorating the quality of life (Figure 3; Adams and Schuler, 2012). Indeed, exercise favors the reduction in body weight and LDL cholesterol (Lee et al., 2013), the increase in HDL cholesterol (Lee et al., 2013), and insulin sensitivity (Borghouts and Keizer, 2000) thus preventing pathologic conditions such as obesity, atherosclerosis, and diabetes (Ruderman and Schneider, 1992). Regular physical exercise decreases blood pressure both at rest and during exercising, thus preventing a hypertensive state (Cornelissen and Smart, 2013). Overall, these findings suggest the effectiveness of PA to reduce cardiovascular risk.

Also, inflammation and endothelial function, which are involved in cardiac diseases, are two important targets of PA. Both preclinical and clinical studies suggest acute and long-term anti-inflammatory effects of exercise by increasing anti-inflammatory cytokines and reducing pro-inflammatory mediators (IL-6 and TNFalpha) in different tissues (Metsios et al., 2020). The anti-inflammatory action is due to increased transcription factor PPAR alpha and the reduction of NFkappaB levels (Santos et al., 2016). Moreover, PA improves endothelial function by reducing reactive oxygen species production and increasing nitric oxide bioavailability (Di Francescomarino et al., 2009; Skrypnik et al., 2014). In particular, in response to myocardial IR injury, PA is protective by activating adrenergic receptors type 3 (β3AR) and increasing the cardiac storage of nitric oxide metabolites (Calvert et al., 2011). Angiogenesis is also induced by repeated exercise through VEGF gene expression and EPCs release (Strehlow et al., 2003; Di Francescomarino et al., 2009). Research in the field is still ongoing to identify other PA targets, which could explain its effects. In this context, mitochondrial function is critical for heart health. Indeed, physical exercise benefits are associated with increased energy expenditure with a high impact on mitochondrial metabolism. In response to exercise, mitochondria increase ATP synthesis rates to address the cell’s metabolic requests (Sato et al., 2019). To this aim, several nuclear and cytoplasmic proteins are activated to induce MQC and recover mitochondrial function. These findings support the proof of concept that exercise could represent a “*mitochondrial medicine for muscle*,” including the heart, by counteracting mitochondrial dysfunction (Memme et al., 2019; Oliveira and Hood, 2019; Islam et al., 2020).

## Exercise and Cardiac Diseases

In combination with traditional therapies, exercise training is considered a therapeutic tool in coronary heart disease being a critical component in the rehabilitation program of patients after a cardiac event (Kachur et al., 2019). Long-term exercise training improves life quality and reduces hospitalization for cardiovascular diseases and cardiac death in patients with heart failure (Belardinelli et al., 2012).

The molecular mechanisms underlying these effects remain to be defined. Several findings point to mitochondria as the target of the adaptative responses of the heart to PA. Indeed, mass spectrometry analysis in healthy hearts from animal models after 54 weeks of moderate treadmill exercise show an increase in mitochondrial protein content with specific reprogramming of the phosphoproteome (Ferreira et al., 2014). However, an adequate exercise training plan also affects mitochondrial function and ameliorates cardiac function in pathologic conditions, such as aging, IR, myocardial infarction, heart failure, diabetic cardiomyopathy (DCM), and doxorubicin-dependent cardiotoxicity.

### Exercise and Aging

Structural and functional changes in the heart occur in aging leading to cardiac dysfunction and a progressive loss of muscle mass and strength, known as sarcopenia (Carter et al., 2015). These changes are due to alterations of different molecular mechanisms (decrease of PI3K/AKT and β-adrenergic receptor signaling, impaired calcium handling, mitochondrial dysfunction,n and increased ROS production) and can be mitigated by regular exercise (Lopez-Otin et al., 2013; Roh et al., 2016). Aging is *per se* associated with alterations in MQC at different steps. A reduction of mitochondrial biogenesis in aging is due to alterations in AMPK, SIRT1, and PGC-1α activation (Kim et al., 2017). Autophagy and autophagic flux are generally decreased in aging hearts, leading to the accumulation of misfolded proteins and dysfunctional organelles. Accordingly, at the morphological level, aging skeletal muscle mitochondria mainly undergo fission, resulting in smaller, and fragmented mitochondrial structures (Joseph et al., 2012). Also, ROS progressively accumulate during aging, due to impairment of mitochondrial oxidative phosphorylation (Shirakabe et al., 2016). Indeed, the aged heartis characterized by a decreased oxidative capacity due to defects in the complexes III and IV of the electron transport chain leading to increased ROS levels. Preclinical studies in aged rats show that regular exercise is cardioprotective by reversing mitochondrial function and quality, oxidative stress, and apoptosis (Carter et al., 2015; Chen et al., 2018; No et al., 2020; Zhang et al., 2020). In particular, exercise increases beta-adrenergic and IGF1 signaling, calcium handling by regulating SERCA activity, and mitochondrial dynamics, by inducing PGC-1alpha (Roh et al., 2016). All these findings suggest the potentiality of exercise to revert cardiac aging in humans.

### Ischemia-Reperfusion Injury

Myocardial ischemia/reperfusion leads to significant cardiac metabolic changes that strongly affect the contractile function (Rosano et al., 2008). These metabolic changes are initially beneficial, allowing the adaptative responses of the heart to the ischemic condition. However, they become chronically detrimental, contributing to the ischemic injury (cardiomyocyte death and contractile dysfunction) perpetuated in the first reperfusion phase (Rosano et al., 2008). During the ischemic period, damage to the mitochondrial electron transport chain leads to oxidative and mitochondrial damage. In the following reperfusion phase, damaged mitochondria worsen cardiomyocyte injury, leading to excessive ROS production, alterations of calcium handling, depolarization, and mitochondrial membrane (Lesnefsky et al., 2017). In this context, the activation of mitophagy is essential to counteract the progression of mitochondrial damage. A novel alternative mitophagy pathway has been recently described and protects the heart against ischemia (Saito et al., 2019). This pathway is based on the action of a multiprotein complex consisting of Ulk1, Rab9, Rip1, and Drp1 (Saito et al., 2019). Ulk1-dependent phosphorylation of Rab9 favors the interaction between Rab9 and Rip1 and the consequent phosphorylation of Drp1, leading to the activation of mitophagy. Thus, manipulations of mitochondrial dynamics are encouraged to increase therapeutic intervention opportunities in response to ischemia/reperfusion.

The analysis of mitochondria isolated from hearts of sedentary and exercise-trained rats suggests that exercise can counteract mitochondrial damage: increases antioxidant enzymes and the expression of anti-apoptotic proteins, reduces ROS production, and release of cytochrome c from mitochondria (Kavazis et al., 2008). These effects favor the development of a protective cardiac mitochondrial phenotype that resists apoptotic stimuli. This protective role of mitochondria also occurs in the heart against IR (Lee et al., 2012). Indeed, exercise training protects mitochondria from IR-induced uncoupling and oxidative damage by increasing the levels of cardiac mitochondrial 4-hydroxynonenal-conjugated proteins and mitochondrial antioxidant enzymes. Also, PA prevented the IR-induced release of cytochrome c from the mitochondria (Lee et al., 2012).

### Myocardial Infarction and Heart Failure

**FIGURE 4:**
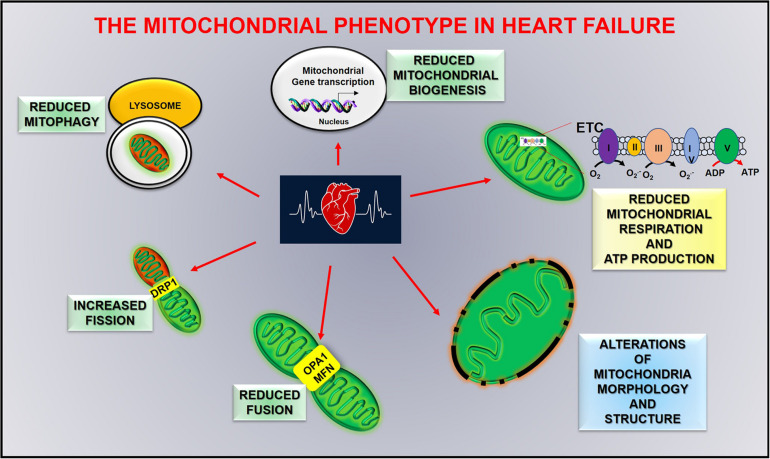
Mitochondrial alterations in heart failure.

Defects in mitochondrial function play a central role in the pathogenesis of myocardial remodeling and heart failure progression, affecting clinical features of heart failure, including skeletal muscle dysfunction, and renal pathologies. The severity of these alterations is strongly associated with the progression of cardiac damage transitioning from physiological hypertrophy to heart failure (Chaanine et al., 2020). In acute myocardial infarction, autophagic flux is impaired and leads to the accumulation of damaged mitochondria, reduced oxygen consumption, and an increase of calcium-induced mitochondrial permeability. However, 8 weeks of exercise training after myocardial infarction counteract such effects. Autophagic flux, mitochondrial bioenergetics, and oxidative capacity are improved in trained mice, and overall cardiac function is ameliorated (Campos et al., 2017). Mitochondrial dysfunction and metabolic alterations worsen progressing to severe systolic dysfunction. In this late stage of cardiac dysfunction (advanced heart failure) mitochondrial morphology and dynamics are severely impaired, as well as fatty acid and glucose metabolism, with an increase of mitochondrial fission proteins (DRP1), a reduction of fusion proteins (OPA1 and MFN) and a downregulation of PGC-1alpha activity (Figure 4; Sabbah, 2020). Exercise reduces such defects by increasing energetic metabolism and autophagy and reducing calcium uptake and ROS production (Campos et al., 2017). This evidence underlines an association between mitochondrial damage and severity of cardiac dysfunction, which allows us to hypothesize that mitochondria could be an early trigger of cardiac damage.

### Diabetic Cardiomyopathy

Diabetic cardiomyopathy involves alterations of energy metabolism (Lorenzo et al., 2013; Nirengi, 2020). The diabetic heart almost exclusively depends on fatty acid degradation to maintain ATP production, which reduces cardiac efficiency (Nirengi, 2020). This causes mitochondrial dysfunction, accumulation of ROS, reduced autophagy, enhanced cell death, and the development of a progressive pro-inflammatory and profibrotic phenotype (Tan et al., 2020). Indeed, pre-clinical studies show a reduction of AMPK activity and an increased expression of mTOR in diabetic hearts from db/db mice, which are associated with the inhibition of autophagy in the heart (Kanamori et al., 2015). mTOR is a key mediator of the insulin signaling pathway and its chronic activation in diabetic hearts suppresses insulin receptor substrate blocking PI3K/Akt signaling and resulting in insulin insensitivity (Suhara et al., 2017). All these conditions lead to cardiac dysfunction and heart failure.

Exercise protects the heart against ROS accumulation during the development of DCM. In a diabetic mouse model, exercise ameliorates blood pressure and systolic dysfunction and improves mitochondrial function by shifting energy metabolism from fatty acid to glucose oxidation (Wang et al., 2020). Accordingly, in a rat model of diabetes, resistance exercise reduces reactive oxygen species production and improves mitochondrial function. In particular, it increases mitochondria numbers, mitochondrial membrane potential, mitochondrial biogenesis, and its regulators (Ko et al., 2018).

### Anthracyclines Dependent Heart Failure

It is well known that cardiac dysfunction could also be induced by the cardiotoxic effect of anticancer drugs like anthracyclines (Gambardella et al., 2017; Tufano et al., 2018; Tocchetti et al., 2020). Several studies suggest mitochondrial dysfunction in doxorubicin-dependent cardiac damage, with alterations of mitochondrial dynamics (Green and Leeuwenburgh, 2002; Ichikawa et al., 2014; Buondonno et al., 2016). Regular exercise can counteract this effect by preventing doxorubicin dependent activation of the apoptotic signaling and alterations in mitochondrial dynamics, including mitophagy (Marques-Aleixo et al., 2018). Based on these findings, PA is now considered a therapeutic tool to address some adverse effects of cancer treatment (Ingram et al., 2010; Furmaniak et al., 2016) and prevent cardiotoxicity (Gilchrist et al., 2019). In this context, a statement from the American Heart Association provides an overview of the existing knowledge in the use of cardiac rehabilitation to cancer patients and survivors and introduces the novel concept of “cardio-oncology rehabilitation” (Gilchrist et al., 2019).

## Conclusion

**FIGURE 5:**
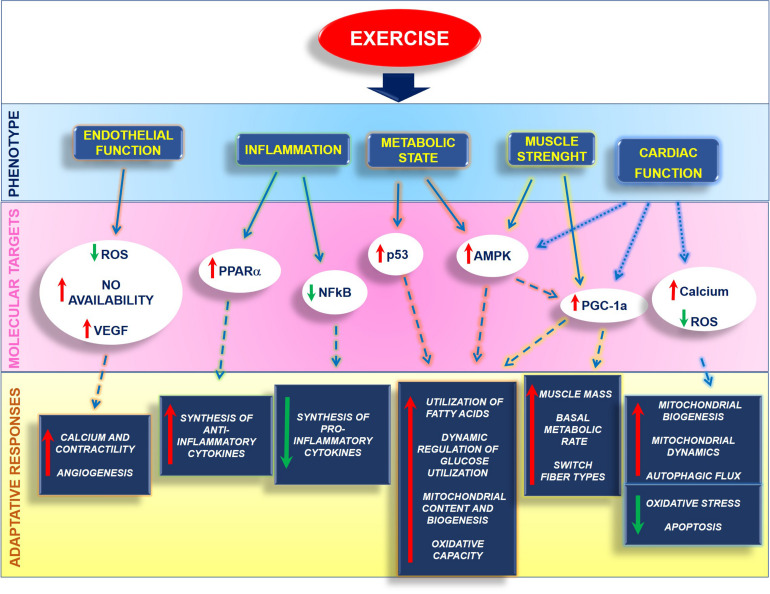
Exercise affects several phenotypes within the cell by activating different molecular mechanisms that orchestrate the adaptative responses of organ and tissues.

Mitochondria are critical players for human health, and their functional integrity is essential for maintaining a well-functioning heart. Metabolic alterations strongly affect cardiovascular diseases, not only as a secondary effect of cardiac damage but also as a trigger of dysfunction. To date, several critical molecules of mitochondria quality control have been identified that could be targeted to ameliorate mitochondrial function, even if a drug that targets explicitly mitochondria has not been generated yet. In this context, exercise represents a non-pharmacologic tool that can ameliorate human health and the quality of life of healthy and ill patients by affecting several cardiac phenotypes and inducing adaptative responses to insults (Figure 5).

Exercise protects endothelium by reducing ROS production and increasing VEGF expression and NO bioavailability, favoring calcium handling and contractility. It also exerts an anti-inflammatory action by inhibiting NFkappaB and activating PPARalpha, thus regulating pro and anti-inflammatory cytokine production. Also, exercise regulates the metabolic state by increasing PGC-1alpha both directly or through the activation of AMPK. This induces the utilization of fatty acids and dynamic regulation of glucose utilization, as well as an increase of mitochondrial function and oxidative capacity. In muscles, including the heart, exercise induces physiological hypertrophy and regulates the switch of fiber types. Also, exercise reduces cardiovascular risk and regulates critical mechanisms of the cardiac mitochondrial machine that allow the recovery of mitochondrial damage and the restoration of the energetic metabolism.

Thus, PA is essential to preserve heart health and reduce the clinical signs associated with energetic cardiac alterations. Therefore, a structured and personalized exercise training plan should be prescribed to everyone, especially older and ill patients. Nevertheless, people’s general trend is toward a sedentary lifestyle increases the prevalence of obesity and associated cardiovascular diseases. To date, the numerous interventions aimed to promote PA are not producing great success since adults, especially older, are reluctant to change their daily routine. More effort from institutions and medical doctors is needed to promote PA, especially to middle-aged adults.

## Author Contributions

DS, ED, and GI conceived and designed the study, collected and analyzed data, wrote the manuscript, and approved the submitted version. All authors contributed to the article and approved the submitted version.

## Conflict of Interest

The authors declare that the research was conducted in the absence of any commercial or financial relationships that could be construed as a potential conflict of interest.
